# A Slight Eulogistic Mistake

**Published:** 1866-04

**Authors:** 


					﻿Editorial.
A SLIGHT EULOGISTIC MISTAKE.
In our enthusiasm, in view of the wonderful progress of our
profession, we are liable to do injustice to the past. In the elo-
quent address of the retiring President, at the late meeting of the
“ Mississippi Valley Association,” we are invited to look “at the
order of exercises, and the lay out of subjects for discussion at
this meeting, and compare it with ten years ago. Then the half
the time of the meeting, was devoted to the pros and cons of am-
algam, or similar nonality ; now we strike infinitely higher etc.”
I have made the comparison, and am convinced that the speak-
er himself must have failed to do so. See vol. IX. p. 237-8,
of the “ Dental Register.” Take, as specimens, three or four of
the questions:
“ How can the color of the natural teeth that require filling, be
most effectually preserved ?”
“ What is the best method of preparing gold for filling teeth ?”
“What are the various pathological conditions of inflamed
dentine; and what circumstances modify its treatment?”
“ What is the modus operand! of arsenic, chloride of zinc, ni-
trate of silver, creosote, tannin, etc., when applied to inflamed
dentine ?”
Now, if at the late meeting, we struck “infinitely higher,” I
am doubtful about our going much deeper. The last question
was partially discussed, and laid over, with a request for members
to consider it, and report at the next meeting, or through the
“Register.” The first article, in vol. X. of the “Register” was
written in accordance with this request, and was read at the “Am-
erican Dental Convention,” simply because it was ready.
And as to “ amalgam,” I have attended all the meetings of the
M. V. A. but one, since 1852, and never heard it discussed, ex-
cept once. Then the old society was asked to rescind a resolu-
tion adopted soon after its organization, which declared the use of
amalgam “unprofessional ;” but the resolution to rescind was
lost, by nearly a unanimous vote. Our worthy President, I am
sure, did not intend to reproach the “ Mother society;” but her
older boys are very jealous of her reputation.	W.

				

